# Exploring the Therapeutic Potential of Cannabidiol in U87MG Cells: Effects on Autophagy and NRF2 Pathway

**DOI:** 10.3390/antiox14010018

**Published:** 2024-12-26

**Authors:** Laura Giannotti, Benedetta Di Chiara Stanca, Francesco Spedicato, Daniele Vergara, Eleonora Stanca, Fabrizio Damiano, Luisa Siculella

**Affiliations:** 1 Department of Experimental Medicine, University of Salento, 73100 Lecce, Italy; benedetta.dichiara@unisalento.it (B.D.C.S.); eleonora.stanca@unisalento.it (E.S.); luisa.siculella@unisalento.it (L.S.); 2 Institute of Polymers, Composites and Biomaterials, National Research Council (IPCB-CNR), 80125 Naples, Italy; 3 Department of Biological and Environmental Sciences and Technologies, University of Salento, 73100 Lecce, Italy; francesco.spedicato@unisalento.it (F.S.); daniele.vergara@unisalento.it (D.V.)

**Keywords:** autophagy, cannabidiol, CBD, NRF2 pathway, oxidative stress, U87MG

## Abstract

Cannabinoids include both endogenous endocannabinoids and exogenous phytocannabinoids, such as cannabidiol (CBD), and have potential as therapeutic agents in cancer treatment due to their selective anticancer activities. CBD exhibits both antioxidant and pro-oxidant effects depending on its concentration and cell types. These properties allow CBD to influence oxidative stress responses and potentially enhance the efficacy of antitumor therapies. In this study, we treated U87MG glioma cells with low dose (1 μM) CBD and evaluated its molecular effects. Our findings indicate that CBD reduced cell viability by 20% (*p* < 0.05) through the alteration of mitochondrial membrane potential. The alteration of redox status by CBD caused an attempt to rescue mitochondrial functionality through nuclear localization of the GABP transcription factor involved in mitochondria biogenesis. Moreover, CBD treatment caused an increase in autophagic flux, as supported by the increase in Beclin-1 and the ratio of LC3-II/LC3-I. Due to mitochondria functionality alteration, pro-apoptotic proteins were induced without activating apoptotic effectors Caspase-3 or Caspase-7. The study of the transcription factor NRF2 and the ubiquitin-binding protein p62 expression revealed an increase in their levels in CBD-treated cells. In conclusion, low-dose CBD makes U87MG cells more vulnerable to cytotoxic effects, reducing cell viability and mitochondrial dynamics while increasing autophagic flux and redox systems. This explains the mechanisms by which glioma cells respond to CBD treatment. These findings highlight the therapeutic potential of CBD, suggesting that modulating NRF2 and autophagy pathways could represent a promising strategy for glioblastoma treatment.

## 1. Introduction

In recent decades, secondary metabolites of *Cannabis sativa* have attracted considerable interest due to their therapeutic potential. Among these secondary metabolites is cannabidiol (CBD), a phytocannabinoid extensively studied in pharmacotherapy due to its chemical properties and biological effects. CBD modulates intracellular redox and inflammatory signals either through the generation of reactive oxygen species (ROS) or by modulation of its membrane receptor activity [1].

The endocannabinoid system plays a key role in numerous pathophysiological processes, making its pharmacological modulation a promising strategy for treating various diseases, including cancer. This system, consisting of the cannabinoid receptors CB1 and CB2 and their endogenous ligands, has been implicated in regulating tumor proliferation, migration, and invasiveness [2].

Among the most chemoresistant tumors, glioblastoma (GBM) is one of the fastest-growing neoplasms with a poor prognosis. Despite great efforts to develop anti-tumor therapeutic strategies and aggressive treatments, including surgery, radiotherapy, and chemotherapy, this tumor is invariably recurrent and leads to death within less than a year of diagnosis [3].

In glioma, particularly in the U343, U251, U87, and T98 cell lines, phytocannabinoids have been shown to act on CB receptors and inhibit tumor growth in animal models by a well-documented mechanism that may also be effective in patients [4,5]. Specifically, cannabidiol has also been shown to modulate the Nuclear Factor Erythroid 2-Related Factor 2 (NRF2) pathway in glioma cells, promoting its activation in a dose-dependent manner [6].

NRF2 transcription factor plays a pivotal role in maintaining redox homeostasis by orchestrating the expression of genes encoding cytoprotective and detoxifying enzymes, such as catalase (CAT), NAD(P)H quinone dehydrogenase 1 (NQO1), and superoxide dismutase (SOD) [7,8,9,10]. The activation of NRF2 is under tight control through its interaction with Kelch-like ECH-associated protein 1 (KEAP1). KEAP1 is part of a ubiquitination complex, which inhibits the activation of NRF2 by targeting it for proteasome-dependent degradation. NRF2 activation involves its dissociation from KEAP1 through two different mechanisms, the canonical KEAP1-dependent and the non-canonical KEAP1-independent pathways [1]. In the canonical activation of NRF2, oxidative stress disrupts the NRF2-KEAP1 complex and prevents the ubiquitination and degradation of NRF2. By contrast, in the non-canonical pathways, KEAP1 uncouples from NRF2 through its interaction with p62, a protein implicated in autophagy, an evolutionarily conserved process involved in the recycling of cytoplasmic organelles, proteins, and macromolecules.

NRF2 is also considered a Janus protein. Under homeostatic conditions, NRF2 activation prevents excessive cellular damage produced by oxidative stress, and thus, it is important in cancer chemoprevention. However, NRF2 activation confers several advantages to cancer cells, including protection against oxidative stress, apoptosis, and senescence, thus promoting cell growth and resistance to chemo- and radiotherapy [11,12,13].

Autophagy has also been shown to interact with NRF2 signaling in glioblastoma models, including U87MG cells. Recent evidence proposed by Kim et al. suggests that CBD modulates autophagy by increasing intracellular ROS levels. In fact, although they did not directly investigate the role of NRF2, they observed that CBD treatment stimulated autophagy in U87MG and U373 glioblastoma cell lines through increased ROS levels [14], which are closely connected to NRF2 activity, as highlighted already.

In U87MG cells, this interplay between ROS, NRF2, and autophagy may mediate dual effects: the removal of damaged cellular components and the regulation of tumor survival. These findings highlight the potential of CBD to target autophagy-related pathways in glioblastoma, requiring further exploration of the molecular details.

Although numerous studies have demonstrated the regulation of NRF2 activity by CBD, there remains limited and inconsistent information regarding the underlying molecular mechanisms. Currently, in fact, several studies report contradictory findings concerning the CBD regulation of the NRF2 pathway and redox status, both in physiological and pathological conditions, including a wide variety of tumors [1]. For instance, Jastrząb et al. reported that in UV-irradiated skin keratinocytes, 1 μM CBD enhances the NRF2 transcriptional activity by increasing the levels of its activators, p21 and p62, and reducing the level of its inhibitor, KEAP1 [15]. Conversely, Bockmann and Hinz reported that 10 μM CBD downregulates the expression of NRF2 and promotes autophagy in human umbilical vein endothelial cells [16]. Apart from the action of CBD on NRF2, in vitro and in vivo studies in various cell lines have also highlighted both the antioxidant and pro-oxidant capacity of CBD caused by its direct modulation of intracellular ROS levels [1].

The U87MG glioblastoma cell line is one of the most widely used in vitro models to study glioblastoma due to its human origin and well-characterized biological properties. It is a valuable tool for studying the aggressive behavior of glioblastoma, its resistance to therapy, and its response to novel treatments, as it recapitulates key molecular and phenotypic features of the tumor. In addition, the availability of extensive molecular and genomic data on U87MG cells makes them a reference model for exploring potential therapeutic strategies and mechanistic pathways in glioblastoma research.

To date, studies exploring the potential properties of CBD in glioma cells are limited, and the molecular mechanisms underlying its effects are poorly understood. Therefore, in the present study, we aimed to characterize the effects of CBD in the U87MG glioma cell line, one of the most widely used in vitro models to study glioblastoma due to its human origin and well-characterized biological properties. The primary objective of the present study was to delve deeper into the intricate mechanisms underlying the effects of CBD, with a specific focus on bridging the knowledge gaps between its multifaceted actions. By exploring the interconnected pathways between CBD and NRF2 activity in relation to oxidative stress, mitochondrial biogenesis, and autophagy, the study aims to provide a more comprehensive understanding of how CBD influences cellular processes and contributes to its potential therapeutic effects, particularly in the context of glioblastoma.

## 2. Materials and Methods

### 2.1. Cell Culture and Treatment

The U87MG cell line (HTB-14, ATCC, Rockville, MD, USA) was incubated in a humidified incubator with 95% air and 5% CO_2_ at 37 °C and cultured in high glucose DMEM (L0101-500, Voden medical instruments, Casorezzo (Mi), Italy) containing 10% (*v*/*v*) fetal bovine serum (FBS, S181H-500, Voden medical instruments, Casorezzo (Mi), Italy), 1% (*v*/*v*) penicillin–streptomycin (P4333, Sigma-aldrich, Milan, Italy) solution, and 2 mM L-glutamine (X0550-100, Voden medical instruments, Casorezzo (Mi), Italy). At approximately 80% confluence, the cells were seeded at different densities depending on the size of the culture dish. After 24 h, the cells were transferred to serum-free conditions and simultaneously treated with CBD or left untreated as a control condition. CBD was from CBDepot (Teplice, Czech Republic). Therefore, the experimental conditions were as follows: control cells treated only with dimethyl sulfoxide (DMSO), used as a vehicle, and 1 μM CBD (CBD).

In our experiments, we used the following inhibitors: cycloheximide (c-7698 Sigma,-aldrich, Milan, Italy) for protein synthesis, MG132 (M7449 Sigma-aldrich, Milan, Italy) for the proteasome, actinomycin D (A9415 Sigma-aldrich, Milan, Italy) for transcription, and chloroquine (50-63-5 Sigma-aldrich, Milan, Italy) for autophagy.

### 2.2. Cell Viability Assay

To assess the viability of U87MG cells after CBD treatment, the MTT (3-(4,5-dimethylthiazol-2-yl)-2,5-diphenyltetrazolium bromide) assay was used. Cells were seeded at a density of 6 × 10^3^ cells/well in a 96-well plate and treated with different concentrations of CBD (1, 5, 10, and 20 μM) for 24 h to evaluate the cytotoxicity of cannabidiol. After determining the optimal CBD effect at the concentration of 1 μM, U87MG cells were seeded in the same manner as previously described and treated for 24, 48, and 72 h to assess the effect of the molecule over time. In both experiments, 20 μL of MTT solution (10 mg/mL) was added to each well at the final concentration of 1 mg/mL and incubated for 4 h at 37 °C [17]. After the incubation, the absorbance was measured with a plate reader at 570 nm.

### 2.3. JC-1 Staining Assay

U87MG cells were seeded in 6-well plates at a density of 9 × 10⁴ and treated under control and CBD conditions for 24 h. At the end of the treatment, the JC-1 dye (sc-364116, Santa Cruz, Heidelberg, German) was used to determine the mitochondrial membrane potential. JC-1 is a fluorescent dye that aggregates or remains monomeric depending on the mitochondrial potential and emits fluorescence at different wavelengths. The dye was reconstituted in DMSO to obtain a 2 mM stock solution and used at a final concentration of 2 µM in PBS. The cells were then washed with PBS to remove any residual medium and incubated with the dye. The incubation time was 20 min at 37 °C with the plate protected from light. After incubation, the JC-1 solution was removed, and the wells were washed with PBS. Finally, images of the samples were captured using a fluorescence microscope (EVOS^TM^ FLoid, Invitrogen, Waltham, MA, USA). JC-1 fluorescence was then read with a plate reader at 590 nm emission for aggregates in mitochondria with high membrane potential, which produce red fluorescence, and at 529 nm emission to assess whether the mitochondrial potential is low, with the dye remaining monomeric and emitting green fluorescence. The red/green ratio was used to assess differences in mitochondrial potential.

### 2.4. Western Blotting Analysis

U87MG cells were seeded at a density of 5 × 10^5^ cells per 100 mm dish for each experimental condition to obtain total, cytosolic, and nuclear cellular protein extracts according to the protocol reported in [18,19]. Total, cytosolic, and nuclear proteins were determined using the Bio-Rad protein assay kit. Lyophilized bovine serum albumin (BSA) was used as a standard. Equal amounts of protein were denatured at 96 °C for 5 min and separated on 10% (*w/v*) SDS gels. The separated proteins were then electrophoretically transferred to a nitrocellulose membrane (Pall, East Hills, NY, USA). Ponceau S staining of blots was carried out to confirm equal protein loading. The filter was then blocked with 2.5% (*w/v*) non-fat dry milk in buffered saline for 1 h at room temperature. The blots were incubated overnight at 4 °C with specific primary antibodies diluted 1:1000. The primary antibodies used were against NRF2 (sc-81342, Santa Cruz, Heidelberg, German), SOD1 (sc-17767, Santa Cruz, Heidelberg, German), CAT (sc-271803, Santa Cruz, Heidelberg, German), GABP (sc-28312, Santa Cruz, Heidelberg, German), Bax (sc-7480, Santa Cruz, Heidelberg, German), Bcl-2 (sc-7382, Santa Cruz, Heidelberg, German), Casp-3 (sc-271028, Santa Cruz, Heidelberg, German), Casp-7 (sc-81654, Santa Cruz, Heidelberg, German), LC3A (#4108, Cell Signaling, Danvers, MA, USA), p62 (sc-48402, Santa Cruz, Heidelberg, German), Beclin-1 (#3495, Cell Signaling, Danvers, MA, USA). Anti-β-actin (sc-47778, Santa Cruz, Heidelberg, German) and anti-Lamin a/c (#4777, Cell Signaling, Danvers, MA, USA) were used for normalization [20,21].

Immune complexes were detected with appropriate peroxidase-conjugated secondary antibodies, anti-mouse (A90-116P) and anti-rabbit (A120-101P), for 1 h at room temperature, and immunoreactive bands were detected using an enhanced chemiluminescence detection kit (#1705061). Densitometric analysis was performed on the blots using the ChemiDoc MP imaging system (Bio-Rad, Hercules, CA, USA).

### 2.5. Real-Time PCR

U87MG cells were seeded in 12-well plates at a density of 3 × 10^4^ cells/well and treated with CBD. After 24 h of treatment, total RNA was extracted using Trizol^TM^ Reagent (ThermoFisher Scientific, Waltham, MA, USA), and the reverse transcription reaction (20 μL) was performed using 1 μg of total RNA, random primers, and the MultiScribe^®^ Reverse Transcriptase (Applied Biosystem, Monza, Italy), according to the manufacturer’s protocols. Quantitative gene expression analysis was performed on a CFX Connect Real-Time system (Bio-Rad, Hercules, CA, USA) using SYBR Green technology (FluoCycle-Euroclone, Milan, Italy). The specificity of PCR products was confirmed by melting curve analysis, and reactions were performed in triplicate on three independent sets of RNA, using *Gapdh* as a normalization gene [20]. The primer sequences used are shown in Table 1.

### 2.6. Cell Transfection and Luciferase Assay

The pGL3-2xARE reporter plasmid was prepared by cloning a DNA fragment containing two binding motifs for the NRF2 transcription factor into the pGL-3 promoter (Promega, Madison, WI, USA) at the KpnI and BglII sites [10]. For transient transfection, 3 × 10^4^ cells were seeded in 12-well plates and transiently transfected with the luciferase reporter construct 24 h later. Transfection was performed using Lipofectamine™ 3000 Transfection Reagent (L3000001 Invitrogen, Waltham, MA, USA) according to the manufacturer’s recommendations. Twenty-four hours after transfection, cells were treated without or with 1 μM CBD. At the end of treatment, cells were lysed, and firefly luciferase activity was measured using the Luciferase Assay System (E1500 Promega, Madison, WI, USA). The control experiment was performed with the pGL3prom empty vector [22]. For transfection normalization, pcDNA3.1/His/lacZ encoding β-galactosidase was used. Variations in β-galactosidase activity between control and treated cells were statistically insignificant, confirming that the experimental conditions did not affect β-galactosidase expression. Luciferase activity values were normalized with respect to protein concentration.

### 2.7. Dapi Staining Assay

Cells were plated at a density of 1 × 10^4^ cells per well in a plate, 24 wells for each experimental condition. At the end of the treatment, the cells were washed with PBS, fixed in 4% paraformaldehyde in PBS pH 7.4 at room temperature for 15 min, and incubated in a 0.1% Dapi solution in PBS at room temperature for 5 min. Images were acquired using a fluorescence microscope (EVOSTM FLoid, Invitrogen, Waltham, MA, USA).

### 2.8. Statistical Analysis

Experiments were conducted independently at least three times, and results were expressed as mean ± standard deviation (SD). Statistical analysis was carried out utilizing GraphPad Prism 9.5 (GraphPad Software, Boston, MA, USA). The data underwent analysis using Student’s *t*-test (with statistical significance defined as *, *p* < 0.05; **, *p* < 0.01; ***, *p* < 0.001; ****, *p* < 0.0001) and one-way analysis of variance (ANOVA) followed by the Bonferroni/Dunn post hoc test (*p* < 0.05). Outliers were neither identified nor treated, and no transformations were applied to the data.

## 3. Results

### 3.1. Effects of CBD on U87MG Glioma Cell Viability

**Figure 1:**
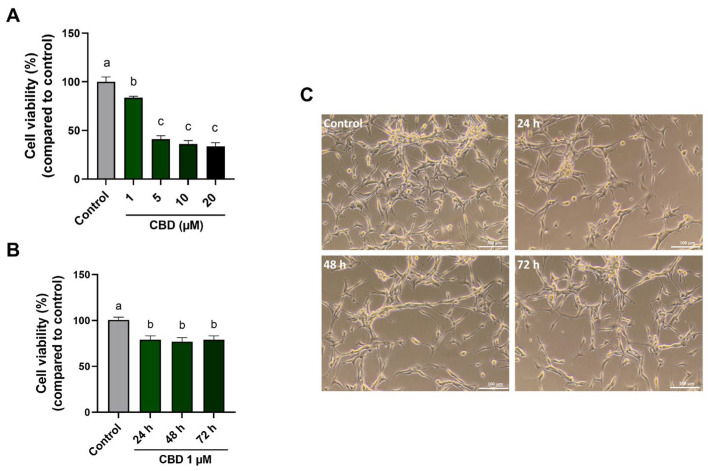
Effects of CBD treatment on cell viability. (**A**) U87MG cells were treated with CBD at 1–5–10–20 μM for 24 h. (**B**) U87MG cells were treated with 1 μM CBD for 24, 48, and 72 h. (**C**) Phase-contrast images of U87MG untreated and treated cells (scale bar 100 μm). The percentage of cell viability in the CBD-treated cells was compared to control cells, and the values are expressed as mean ± SD. Experiments were repeated three times independently (*n* = 3). The results were expressed as a percentage relative to the control group, which was set as the reference at 100%. Samples bearing different letters differ significantly (*p* < 0.05).

To evaluate the cytotoxicity of CBD on the cellular viability of the U87MG cell line, the MTT assay was performed. Compared to untreated cells, treatment with 1 μM CBD for 24 h induced a slight but significant decrease (~20%) in cell viability (Figure 1A). At concentrations of 5, 10, and 20 μM, a more significant reduction in cell viability was observed (55–60%). Based on these data, the concentration of 1 µM was identified as the minimum statistically significant dose affecting viability and was therefore used in our experiments. Subsequently, the time-dependent (24, 48, 72 h) effect of 1 μM CBD on cell viability was also evaluated (Figure 1B). The results of this experiment showed no differences in cellular vitality after 24 h of treatment. Phase contrast images of U87MG cells treated with 1 µM CBD on different days in the time course experiment are shown in Figure 1C. Images indicate that there are no differences in cell growth over time, confirming the findings previously described. Therefore, the following experiments in this work referred to the dose–time of 1 μM for 24 h for CBD treatment.

### 3.2. Effects of CBD on Mitochondrial Functionality and Biogenesis

**Figure 2:**
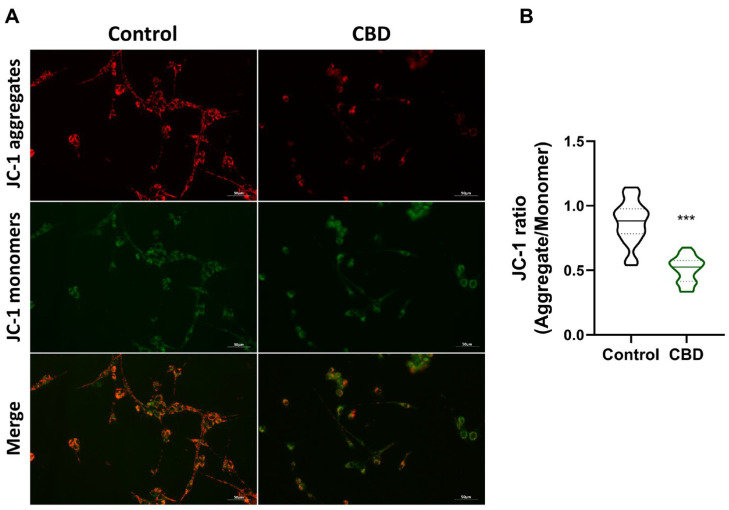
Fluorescence detection of JC-1 in U87MG cells after CBD treatment. (**A**) Fluorescence microscope images of U87MG cells in Control and CBD conditions (scale bar 50 µm). (**B**) Single-cell fluorescence level analysis (red/green ratio) for at least 40 cells from three different images per condition. Results are expressed as mean ± standard deviation (SD). Experiments were repeated three times independently (*n* = 3). *** *p* < 0.001 compared with control.

We hypothesized that exposure to CBD can compromise U87MG viability through mitochondrial function. The JC-1 assay experiment was set up to monitor mitochondrial membrane potential (MMP) by fluorescence microscopy (Figure 2A). The assay involves double fluorescent staining of mitochondria by JC-1, either as green fluorescent J-monomers or as red fluorescent J-aggregates. As shown in Figure 2A, the control sample exhibits JC-1 predominantly in the form of red fluorescent aggregates, indicative of intact MMP. In contrast, the sample treated with CBD displays a significantly reduced presence of red fluorescent aggregates. This observation is further supported by the quantification of fluorescence levels presented in Figure 2B. These results demonstrate that CBD treatment induces an alteration in MMP, consistent with the establishment of an oxidative stress condition triggered by CBD exposure.

**Figure 3:**
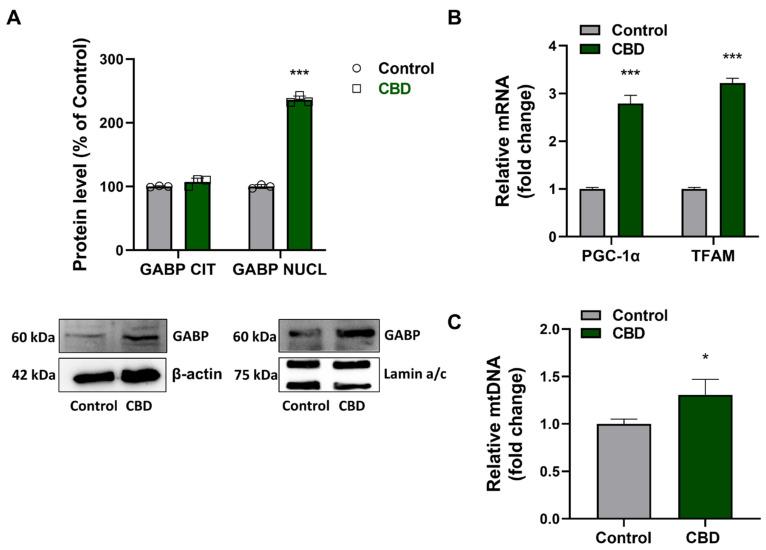
Effects of CBD on mitochondrial biogenesis. (**A**) Analysis of distribution of the transcription factor GABP at the cytoplasmic and nuclear levels. The GABP level was normalized with respect to β-actin or Lamin a/c. The results were expressed as a percentage relative to the control group, which was set as the reference at 100%. (**B**) Quantification of PCG-1α and TFAM mRNA and (**C**) mtDNA. The results were expressed by normalizing the values to the control group, whose mean value was set to 1. Data are normalized to the internal standard GAPDH mRNA or genomic *NCOA3* gene. Results are expressed as mean ± SD. Experiments were repeated three times independently (*n* = 3). * *p* < 0.05 and *** *p* < 0.001 compared with control.

Nuclear respiratory factor 2 (GABP) is a transcription factor activating the expression of genes coding for proteins involved in mitochondrial biogenesis, electron transport, oxidative phosphorylation, and mitochondrial biogenesis. U87MG cells were seeded and treated without (control) or with 1 µM CBD as described previously. After 24 h, Western blotting was performed using cytosolic and nuclear protein extracts. By comparing the GABP content in cytosolic and nuclear protein extracts, results showed that CBD induced nuclear translocation of GABP (Figure 3A).

This finding was supported by the abundance of the mRNA of GABP target genes coding for the transcription factors PGC-1α and TFAM (Figure 3B). Moreover, when compared to control, real-time PCR experiments indicated an increase in mitochondrial DNA (mtDNA) in CBD-treated cells (Figure 3C), suggesting a dynamic remodeling of the mitochondrial network and a consequent regulation of mitochondrial biogenesis.

### 3.3. Effects of CBD on Apoptosis Pathway

**Figure 4:**
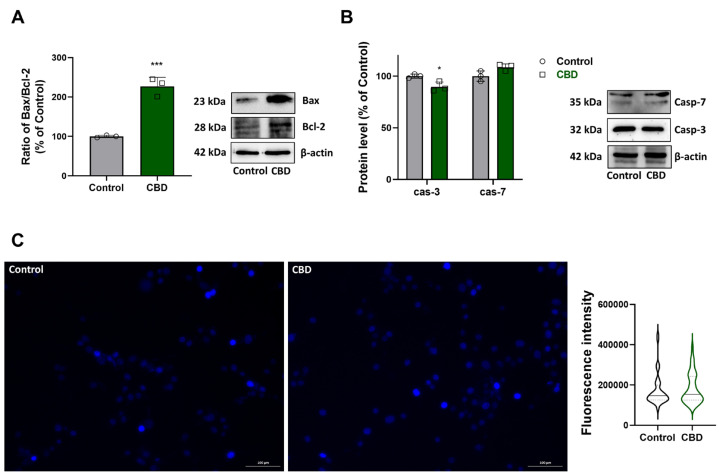
Effects of CBD on the apoptosis pathway. Western blot and densitometric analysis of bands, (**A**) Bax and Bcl-2. (**B**) Casp-7 and Casp-3. Values represent relative optical density after normalization to β-actin expression. The results were expressed as a percentage relative to the control group, which was set as the reference at 100%. Results are expressed as mean ± SD. Experiments were repeated three times independently (*n* = 3). * *p* < 0.05 and *** *p* < 0.001 compared with control. (**C**) DAPI staining in control and CBD conditions (scale bar 100 µm) and graph of single cells fluoresce for at least 60 cells per condition from 3 photos per condition.

The results reported in the previous paragraph highlighted an alteration of mitochondrial functionality that could trigger a cell death process after treatment with CBD. Therefore, we examined the Bax/Bcl-2 ratio and the levels of Caspase 3 (Casp-3) and Caspase 7 (Casp-7). As shown in Figure 4A, according to the Bax/Bcl-2 ratio determination, an increase in the expression of Bax relative to Bcl-2 has been observed in CBD condition compared to control, suggesting the activation of apoptosis. Despite this imbalance, we did not detect the activation of Casp-3 and Casp-7 (Figure 4B), as suggested by the absence of the corresponding cleaved and activated form of the enzymes. Moreover, as can be observed from DAPI-staining images in Figure 4C, there is no significant difference in chromatin condensation between the control and CBD conditions, which indicates the absence of apoptotic nuclei after CBD treatment.

Apoptosis and autophagy are two distinct yet interconnected processes in cellular turnover, with shared regulatory components and stress-induced pathways [23]. On the basis of previous findings about mitochondrial alteration, we explored the effect of cannabidiol on mitochondrial renewal by the autophagy process.

**Figure 5:**
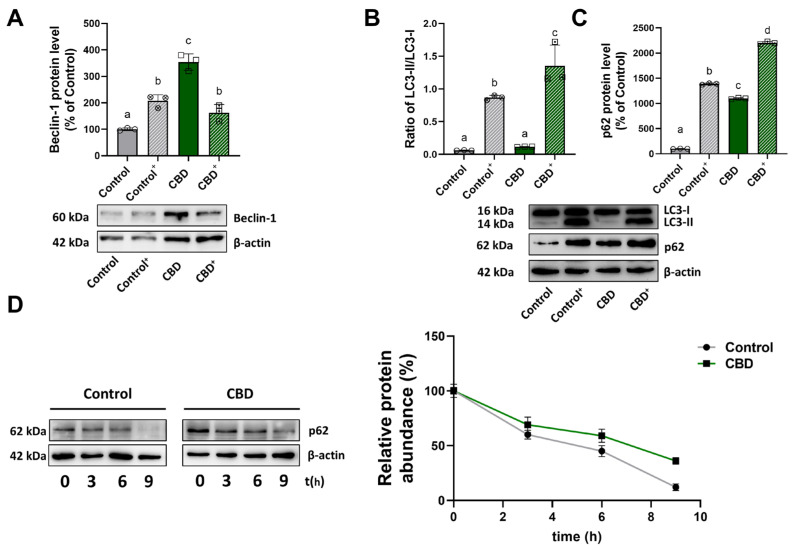
Effect of CBD treatment on autophagic flux. Analysis by Western blotting of Beclin-1 levels (**A**), the ratio of LC3-II/LC3-I (**B**), and p62 levels (**C**). (**D**) Half-life of p62 in the presence and absence of chloroquine inhibitor, under control and CBD conditions. Values were normalized to β-actin expression. Results are expressed as mean ± SD. The results, except for the ratio, were expressed as a percentage relative to the control group, which was set as the reference at 100%. Experiments were repeated three times independently *(n* = 3), and samples bearing different letters differ significantly (*p* < 0.05).

Using 50 μM chloroquine, an inhibitor of the late phase of autophagy, we investigated the expression levels of Beclin-1, involved in the initiation of the autophagic process, LC3-II, associated with the autophagosome formation, and p62, which plays a role in autophagic flux. U87MG cells were seeded under four conditions: control and CBD (untreated and CBD-treated cells), control^+^ (cells treated with chloroquine alone), and CBD^+^ (cells treated with CBD and chloroquine). The inhibitor was added 4 h before the end of CBD treatment. The obtained results indicated that an increase in Beclin-1 protein levels was observed in the CBD condition compared to the control (Figure 5A), suggesting that CBD stimulates the initiation of autophagy. This increase agreed with the observed rise in the LC3-II/LC3-I ratio (Figure 5B), confirming that CBD activates autophagy by enhancing autophagosome formation. Even in the presence of chloroquine and CBD (CBD^+^), the levels of Beclin-1 remain elevated, as those observed with CBD treatment alone. These differences observed between control and CBD suggest that CBD could stimulate the autophagic process, a crucial cellular mechanism for cleaning and recycling cellular components.

Analysis of LC3-II protein levels alone is not sufficient to describe the autophagic process. An alternative method to evaluate autophagic flux is to measure the p62 level and stability. As shown in Figure 5C, p62 protein levels are increased in CBD-treated cells compared to control cells.

Analysis of the p62 half-life (Figure 5D) showed that in the control condition, rapid degradation of the p62 protein occurs, suggesting an active and functional autophagic flux in which the protein is readily cleared as part of the normal cellular turnover process.

On the other hand, in CBD samples, p62 degradation was significantly slowed down, indicating a possible alteration of autophagic flux. The data on the p62 protein contrasted with those of Beclin-1 and LC3, suggesting that p62 could be involved in cellular processes other than autophagy, such as in the oxidative stress response.

### 3.4. CBD Regulates the Expression of NRF2 and Its Target Genes

**Figure 6:**
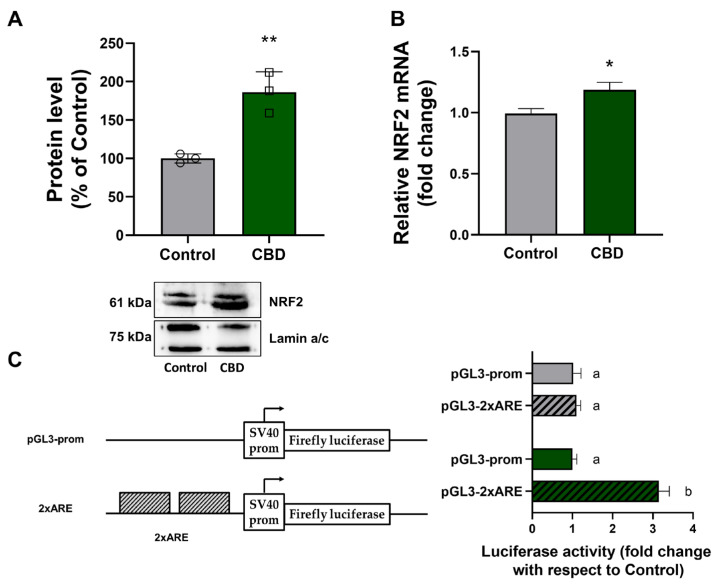
CBD increases NRF2 expression in U87MG cells. (**A**) Nuclear NRF2 protein level in U87MG cells was determined by Western blot and densitometric analysis. In the graph, values represent relative optical density after normalization to Lamin a/c expression. The results were expressed as a percentage relative to the control group, which was set as the reference at 100%. (**B**) NRF2 mRNA abundance was determined by Real-Time PCR using GAPDH as an internal standard. Results are expressed as mean ± SD. Experiments were repeated three times independently *(n* = 3). * *p* < 0.05 and ** *p* < 0.01 compared with control. (**C**) In vitro luciferase assay was performed with the pGL3 prom vector and the pGL3-2xARE construct. The histograms show the relative luciferase activity measured after pGL3-prom or pGL3-2xARE transfection into U87MG cells treated with CBD for 24 h. Luciferase activity values are reported as fold induction relative to the activity determined in the control, represented by untreated cells transfected with pGL3-prom or pGL3-2xARE vector. Results are expressed as mean ± SD. The results were expressed by normalizing the values to the control group, whose mean value was set to 1. Experiments were repeated three times independently (*n* = 3), and samples bearing different letters differ significantly (*p* < 0.05).

Based on a non-canonical pathway between p62 and NRF2, we evaluated the effect of 1 μM CBD on the expression of NRF2 in U87MG cells at the protein and mRNA levels by Western blotting and real-time PCR, respectively. As shown in the histograms in Figure 6, after treatment with CBD for 24 h, both nuclear protein level (Figure 6A) and mRNA abundance (Figure 6B) of NRF2 increased with respect to those observed in untreated cells (control).

To investigate whether CBD treatment could affect NRF2 transactivation in cells, a luciferase reporter assay was performed using the pGL3-2xARE plasmid. This construct consists of two tandem copies of NRF2 binding sites (ARE) upstream of the SV40 promoter (Figure 6C). Firefly luciferase (FL) expression is dependent on NRF2 activation induced by CBD stimulation. As shown in Figure 6C 1 μM CBD treatment did not alter FL activity in cells transfected with the empty pGL3prom vector. In contrast, after transfection with PGL3-2xARE plasmid, CBD treatment led to a 3.2-fold increase in FL activity compared to control.

**Figure 7:**
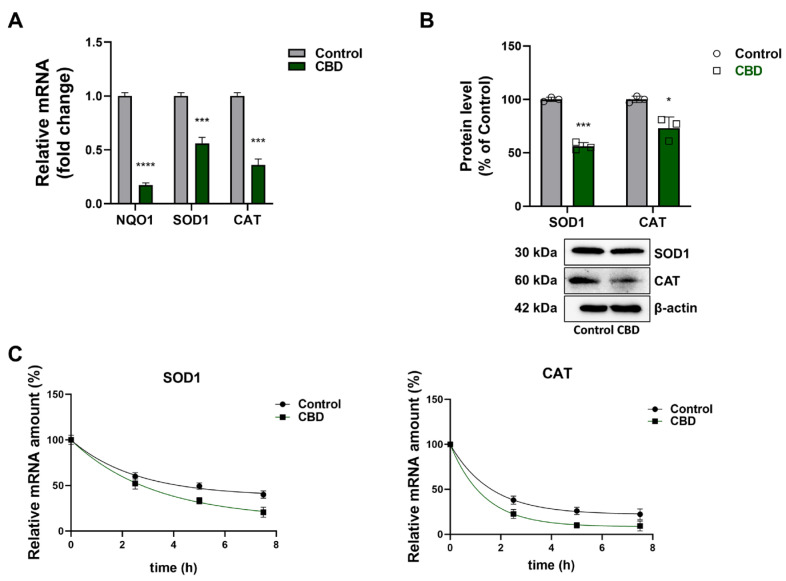
Effect of CBD on NRF2 target gene expression levels. (**A**) Analysis of SOD1, CAT, and NQO1 mRNA abundance normalized against the internal standard GAPDH mRNA. The results were expressed by normalizing the values to the control group, whose mean value was set to 1. (**B**) Western blot and densitometric analysis with values representing relative optical density after normalization to β-actin expression. (**C**) Half-life of SOD1 e CAT mRNA after normalization to 18S rRNA. Results are expressed as mean ± SD. The results were expressed as a percentage relative to the control group, which was set as the reference at 100%. Experiments were repeated three times independently (*n* = 3). * *p* < 0.05, *** *p* < 0.001 and **** *p* < 0.0001 compared with control.

Therefore, the expression of NRF2 target genes was analyzed in CBD-treated cells and in control cells. Contrary to expectations, the mRNA abundance of three NRF2 target genes, *Nqo1*, *Sod1,* and *Cat*, in U87MG cells treated with CBD was reduced by approximately 50–80% compared to control cells (Figure 7A). This finding was confirmed by densitometric analysis of the Western blotting, the expression levels of SOD1 in CBD-treated cells were decreased compared to control, whereas the CATs were constant (Figure 7B).

To explain the contradictory data of reduced expression of NRF2 target genes versus the increased NRF2 transcriptional activity, the decay (half-life) of SOD1 and CAT mRNA was evaluated in CBD-treated cells and in the control (control). As reported in Figure 7C, the black circles represent the time-dependent decrease in SOD1 and CAT mRNA in the control (half-life 2.23 h and 1.1 h, respectively). In CBD-treated samples (green squares), a faster reduction of mRNA was observed, suggesting that CBD treatment accelerated mRNA turnover for these genes compared to controls (half-life 1.76 h and 0.92 h, respectively). Based on these findings, CBD not only reduces SOD1 and CAT protein levels but also produces SOD1 and CAT mRNA abundance by affecting their stability.

### 3.5. Effects of CBD on NRF2 Stability

**Figure 8:**
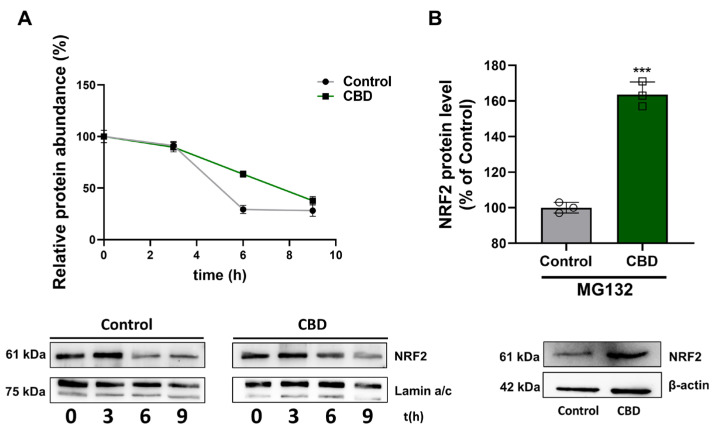
Effects of CBD on NRF2 stability. (**A**) NRF2 half-life measurement after treatment with 100 μM cycloheximide at 0, 3, 6, and 9 h. (**B**) U87MG cells were pretreated with 20 μM MG132, an inhibitor of the ubiquitin–26S proteasome system. NRF2 levels were normalized with respect to Lamin a/c or β-actin. Results are expressed as mean ± SD. The results were expressed as a percentage relative to the control group, which was set as the reference at 100%. Experiments were repeated three times independently (*n* = 3). *** *p* < 0.001 compared with control.

The increased expression levels of NRF2 and the decreased expression of its target genes led us to evaluate the NRF2 stability in U87MG cells after treatment with CBD. Therefore, two parallel experiments were set up with cycloheximide, an inhibitor of protein synthesis, and MG132, an inhibitor of the ubiquitin–proteasome system. U87MG cells were treated with 1 µM CBD for 24 h. A 100 µg/mL amount of cycloheximide was added in the medium, then a time course of the nuclear NRF2 level was established at 0, 3, 6, and 9 h of treatment. A control experiment was performed with CBD-untreated cells. The results reported in Figure 8A showed stabilization of nuclear NRF2 protein in cells treated with CBD with respect to control. The apparent half-life of NRF2 was 7.6 h in CBD-treated cells compared to approximately 5.8 h in control cells (Figure 8A). In the second experiment, the proteasome inhibitor MG132 (20 µM) was added to the medium one hour before the end of CBD treatment, followed by total protein extraction. In the presence of MG132, CBD treatment caused a significant increase in NRF2 amount compared to control (Figure 8B). These findings suggest that CBD treatment promotes NRF2 stabilization in U87MG cells by reducing its degradation rate, likely through modulation of the ubiquitin–proteasome system, as supported by the observed increase in NRF2 levels in the presence of MG132.

## 4. Discussion

Cannabinoids include a large group of organic molecules, both endogenous, such as endocannabinoids, and exogenous, such as phytocannabinoids, extracted from the plant *Cannabis sativa* L. The latter group includes CBD and Δ9-tetrahydrocannabinol, which are widely used in various research and clinical fields. Phytocannabinoids have shown selective anticancer activity in many tumor cell lines, influencing proliferation, differentiation, and cell death, and a possible role as adjuvants in many antitumor therapies has been proposed [24].

CBD, with both antioxidant and pro-oxidant properties, is capable of altering redox-sensitive molecular pathways with significant effects on cell redox status [1]. Furthermore, the multidirectional effects of CBD are also attributed to its chemical structure, with a direct modulatory effect on redox status, resulting primarily from the hydroxyl groups and the pentyl chain in the phenolic ring [1]. The CBD antioxidant/pro-oxidant properties depend on the concentration used and cell specificity, making it a promising candidate for the development of new approaches in the pharmacotherapy of oxidative stress-related diseases.

The antioxidant action of CBD has been demonstrated in various cell types. In mouse hippocampal neurons, CBD administration increased glutathione levels and SOD1 and glutathione peroxidase activities [25], whereas in skin keratinocytes, CBD reduces UV-induced ROS generation [26]. The use of 16 μM CBD promotes high mitochondrial ROS production in human monocytes and induces apoptosis in a dose-dependent manner [27]. A study conducted by Jeong et al. in colorectal cancer cells showed that the use of CBD (4 μM) induces autophagy by increasing mitochondrial dysfunction mediated by ROS overproduction [28]. In addition, a CBD concentration of 25 μM in human THP-1 monocytes promotes the expression of cyclooxygenase 2, which generates nitric oxide responsible for oxidative stress and pro-inflammatory activity associated with pathologies [29].

In gastric cancer cells, the use of CBD at high concentrations up to 120 μM leads to cell cycle arrest in the G0–G1 phase, suggesting that CBD may have therapeutic effects on this type of cancer [30]. Our results showed that in U87MG cells, a low dose of CBD (1 μM) caused a cell viability reduction with the alteration of MMP.

GABP has been considered the master transcription factor in mitochondrial biogenesis and function by modulating the expression of genes for mitochondrial proteins and co-regulators. Yang and coauthors reported that conditional GABP knock-down in primary mouse embryonic fibroblast reduced mitochondrial mass, ATP production, oxygen consumption, and mitochondrial protein synthesis without any effects on apoptosis and on mitochondrial morphology and membrane potential [31]. Our findings demonstrated that, in U87MG cells, CBD treatment caused cytosolic-nuclear shuttling of GABP, as well as the induction of *Tfam*, a GABP-target gene coding for the mitochondrial transcription factor TFAM. In agreement with the TFAM role in the unwinding process during mitochondrial replication [32], the observed increase in TFAM expression was also paralleled by a mtDNA increment in CBD-treated cells. Moreover, the augmented expression of another GABP-target gene coding for PGC-1α, a crucial co-factor involved in mitochondrial biogenesis, was also observed after CBD treatment as a response to stress conditions, helping to mitigate ROS levels and supporting mitochondrial function by increasing the production of detoxifying enzymes [33]. Altogether, these findings supported the notion of attempts to restore the bioenergetic capacity of cells by inducing the biogenesis of mitochondria after their alteration by CBD.

The survival of cancer cells depends on their ability to evade apoptotic and autophagic mechanisms that lead to cell death. Anti-apoptotic proteins of the Bcl-2 family, such as Bcl-2 and Bcl-xL, are often overexpressed in tumors and are also known for their anti-autophagic capabilities. They inhibit apoptosis by binding to pro-apoptotic Bax or Bak proteins and suppress autophagy by binding to Beclin-1, which is required for autophagosome initiation [34,35]. Apoptosis and autophagy, each involving different proteins, are cross-talking processes crucial for cell fate [30]. Autophagy serves as a survival pathway to suppress apoptosis, but in other cases, it can lead to cell death in cooperation with apoptosis [31,36].

Our results showed that low-dose CBD induced an increase in the expression of the pro-apoptotic protein Bax compared to the anti-apoptotic protein Bcl-2. This imbalance between Bax and Bcl-2 is a classic indicator of the induction of apoptosis, the process of programmed cell death. However, despite this imbalance, the activation of Casp-3 and Casp-7, key enzymes in the execution of apoptosis, were not observed. The lack of Casp-3 and Casp-7 activation indicates that CBD triggered the alteration of MMP, as revealed by the JC-1 assay. In fact, it has been reported that a progressive decline in MMP results in a reduction of ATP production, ultimately leading to cell death that is independent of caspase activity [37,38,39].

CBD could stimulate the autophagic process, which is essential for removing and renewing damaged mitochondria. This effect could be the key to understanding how CBD impacts cellular viability and functionality, especially in pathological models of cancer.

Beclin-1, LC3, and p62 are the most analyzed markers among the proteins involved in autophagy. In particular during autophagy, Beclin-1 plays a pivotal role in the initiation of autophagy by promoting the formation of autophagosomes-membrane-bound structures that engulf and degrade damaged cellular components [40]; the cytosolic form of LC3 (LC3-I) is conjugated to phosphatidylethanolamine to form LC3-phosphatidylethanolamine conjugate (LC3-II), associated with the formation of autophagic membranes; and p62 is both an autophagy adaptor protein and a substrate for degradation in the autolysosome [41]. Moreover, since p62 is located on mitochondria, it contributes to mitochondrial renewal by driving non-functional mitochondria. Thus, a standard protein pattern of activated autophagy predicts an increase in Beclin-1 and LC3-II protein and a decrease in p62 because of its degradation in autolysosomes [42,43]. Our findings demonstrated an increase in Beclin-1 and LC3-II but also a strong increment of p62 in CBD-treated cells. The increase in Beclin-1 in response to CBD, along with the elevated LC3-II/LC3-I ratio, indicates that CBD induces the autophagic process. However, our findings indicated that p62 accumulated in both CBD and CBD^+^ (cells treated with CBD and chloroquine), and this increment may be related to the role of p62 in other cellular processes besides autophagy. Recent reports indicate that p62 may also regulate cancer cell metabolism through several mechanisms. It has been shown that phosphorylated S351-p62 can continuously activate NRF2, thereby reprogramming glucose and glutamine metabolism, which could significantly promote cancer cell survival [44]. It has also been shown that increased levels of p62 sequester KEAP1 from its function in the degradation of the stress-responsive transcription factor NRF2, leading to the stabilization of NRF2 and its nuclear translocation [45].

Research suggests that CBD may affect the NRF2 pathway in glioblastoma cells by activating NRF2 and increasing the expression of antioxidant and detoxification enzymes. This activation may protect cells from oxidative stress and inflammation, thus contributing to cancer progression [6]. Studies on other cell lines, such as UVA/B irradiated skin keratinocytes, have shown that CBD-induced decrease in the levels of NRF2 inhibitors (KEAP1, BACH1) and an increase in the level of NRF2 activators (KAP1, p21, p62) [15], which contributes explaining the activity of the NRF2 pathway following stimuli, and also involvement of NRF2 in mitochondrial biogenesis and mitochondrial quality control has been reported [46].

Based on what has been reported in the literature, we investigated NRF2 expression levels in the glioblastoma cells after treatment with CBD because this molecular mechanism presents limited understanding. The findings obtained from our experiments showed that, in U87MG cells, low dose (1 μM) CBD leads to NRF2 activation, as indicated by the increased nuclear NRF2 levels and transcriptional activity, through a protein stabilization mechanism. Paradoxically, the expression of NRF2 target genes (*Nqo1*, *Sod1*, *Cat*), both at the protein and mRNA levels, was reduced in CBD-treated cells, implying a reduced ROS detoxification capacity, contributing to MMP alteration. Besides activation of NRF2, treatment with CBD determined a reduced SOD1 and CAT mRNA stability, thus explaining the discrepancy observed between the augmented NRF2 activation and the reduced expression of NRF2 target genes.

Our findings seem aligned with those of Rybarczyk et al., who reported that CBD can modulate the dormant state of cancer stem cells, which are typically resistant to conventional treatments. By activating Nrf2, CBD promotes cellular resilience and modulates oxidative metabolism, maintaining cells in a quiescent state and reducing uncontrolled proliferation. These findings suggest that CBD, through the Nrf2 pathway, has the potential to modulate cellular quiescence in the tumor context, in agreement with the results of our study in the U87MG glioblastoma cell line [6].

However, this study has several limitations that should be considered when interpreting the results. First, all experiments were performed in vitro using the U87MG cell line, a representative model of human glioblastoma. Even so, in vitro conditions cannot fully replicate the complexity of the tumor microenvironment in vivo, limiting the ability to extrapolate the results to the clinical context. Finally, although the molecular mechanisms involved have been explored, further investigation is required to fully understand the interactions between NRF2 pathways, autophagy, and oxidative stress induced by CBD, particularly given the complexity of the cellular responses observed. Future studies could benefit from a more detailed analysis of different CBD concentrations and cell lines and then translated to in vivo models.

In conclusion, these combined molecular effects suggest that CBD, administered at low doses to glioblastoma cells, could cause oxidative stress, leading to mitochondrial damage that would be repaired through the activation of autophagy and the increase in NRF2 levels, inducing a state of quiescence in the glioblastoma.

**Figure 9:**
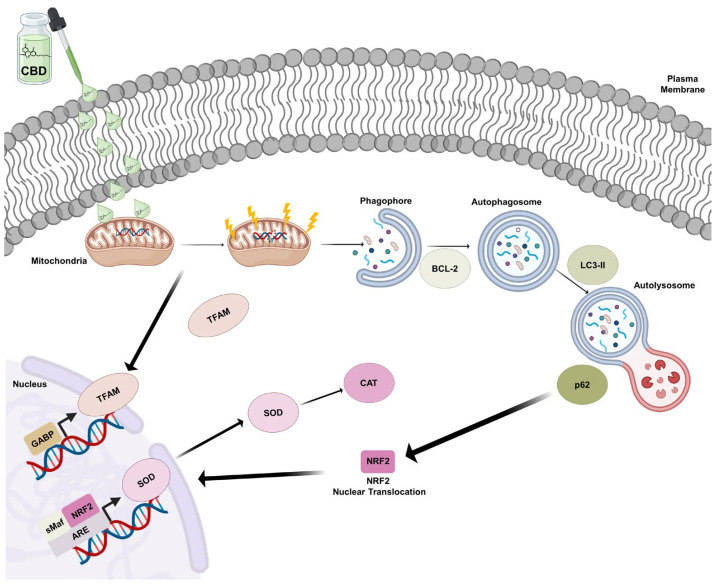
Summary diagram of CBD effects on U87MG cells. CBD (1 μM) reduces the viability of U87MG cells by altering mitochondrial potential and increasing autophagic flux. Additionally, it enhances NRF2 and p62 levels, modulating the redox system and sensitizing the cells to treatment.

Therefore, CBD may contribute to slowing down glioblastoma progression and aid in the development of multi-target agents acting on the NRF2 mitochondrial biogenesis–autophagy axis (Figure 9).

## 5. Conclusions

Our study demonstrates that low-dose CBD treatment (1 μM) in U87MG glioblastoma cells stimulates the autophagy process, which is essential for mitochondrial renewal, contributing to an increase in mitochondria with altered membrane potential. Moreover, CBD-treated U87MG cells present an abnormal activation of the NRF2 pathway, reducing the expression of antioxidant target genes and consequently altering mitochondrial integrity. These molecular effects suggest that CBD could have therapeutic repercussions or be useful in the development of multi-target agents acting on the NRF2 mitochondrial biogenesis–autophagy axis. Further studies are needed to clarify the molecular mechanisms acting between CBD and glioblastoma cells. Future research should focus on validating these findings through comprehensive in vivo studies, investigating the synergistic potential of therapeutic combinations, and exploring translational approaches to facilitate clinical application. This will be essential to advance our understanding and pave the way for potential therapeutic interventions.

## Figures and Tables

**Table 1:** Oligonucleotides used for real-time PCR analysis.

	Sequences (5′-3′)	Accession Number
GAPDH	F: ATGGCCTTCCGTGTCCCCAC R: ACGCCTGCTTCACCACCTTC	NM_014364.5
NRF2	F: GGAACAGCAGTGGCAAGATCTC R: GCAAGGCTGTAGTTGGTGCTCA	NM_003204
NQO1	F: CCTGCCATTCTGAAAGGCTGGT R: GTGGTGATGGAAAGCACTGCCT	NM_000903
SOD1	F: CTCACTCTCAGGAGACCATTGC R: CCACAAGCCAAACGACTTCCAG	NM_000454
CAT	F: GTGCGGAGATTCAACACTGCCA R: CGGCAATGTTCTCACACAGACG	NM_001752
PGC1-α	F: CCAAAGGATGCGCTCTCGTTCA R: CGGTGTCTGTAGTGGCTTGACT	NM_013261.5
TFAM	F: GTGGTTTTCATCTGTCTTGGCAAG R: TTCCCTCCAACGCTGGGCAATT	NM_003201.3
mtDNA	F: CACCCAAGAACAGGGTTTGT R: TGGGCCATGGGTATGTTGTTA	MG817368.1
18S	F: GTTGGTTTTCGGAACTGAGGC R: GTCGGCATCGTTTATGGTCG	9C3H_S2
NCOA3	F: CCTCTGGGCTTTTATTGCGAC R: CCCCTCAACACTGCTCTCCTTAC	NM_181659.3

## Data Availability

Data are contained within the article.
